# Using CRISPR knock-in of fluorescent tags to examine isoform-specific expression of EGL-19 in *C. elegans*

**DOI:** 10.17912/micropub.biology.000858

**Published:** 2023-09-07

**Authors:** Kara McDonald, Kerry Larkin, Daniel J Dickinson, Andy Golden, Xiaofei Bai, Ryan Doonan

**Affiliations:** 1 Glow Worms Stream, Freshman Research Initiative, College of Natural Sciences, The University of Texas at Austin, Austin, Texas, United States; 2 Laboratory of Biochemistry and Genetics, National Institute of Diabetes and Digestive and Kidney Diseases, National Institutes of Health, Bethesda, Maryland, United States; 3 Department of Cell Biology, Yale University, New Haven, Connecticut, United States; 4 Department of Molecular Biosciences, The University of Texas at Austin, Austin, Texas, United States; 5 Department of Biology, University of Florida, Gainesville, Florida, United States

## Abstract

L-type voltage-gated calcium channels (VGCCs) regulate calcium influx and excitation-contraction coupling in many types of muscle cells. Thus, VGCC mutations can cause skeletal and cardiac muscle diseases in humans, such as Duchenne muscular dystrophy and Timothy syndrome. To better understand the genetics and native expression of VGCCs, we have chosen to use the microscopic roundworm, *C. elegans* . The *egl-19* locus is the sole L-type VGCC gene and it encodes three distinct isoforms (a, b, and c). Isoform c is curious because the protein is truncated, lacking the transmembrane domains that form the physical calcium channel. In this study, we have characterized *egl-19* expression using CRISPR/Cas9 genome editing to ‘knock-in’ fluorescent tags of differing colors, allowing us to distinguish the expression pattern of each isoform. Not surprisingly, we found that EGL-19 is expressed in all types of muscle. In addition, we provide evidence that the truncated c isoform is expressed. Finally, although we find evidence that specific isoforms can have unique subcellular distributions, we also observed some expression patterns that appear to be artifacts. Overall, our results show interesting patterns of *egl-19* expression, but also highlight the need for caution when interpreting expression of reporter genes even when they represent endogenous tags.

**Figure 1. Comprehensive analysis of EGL-19 isoform expression:**
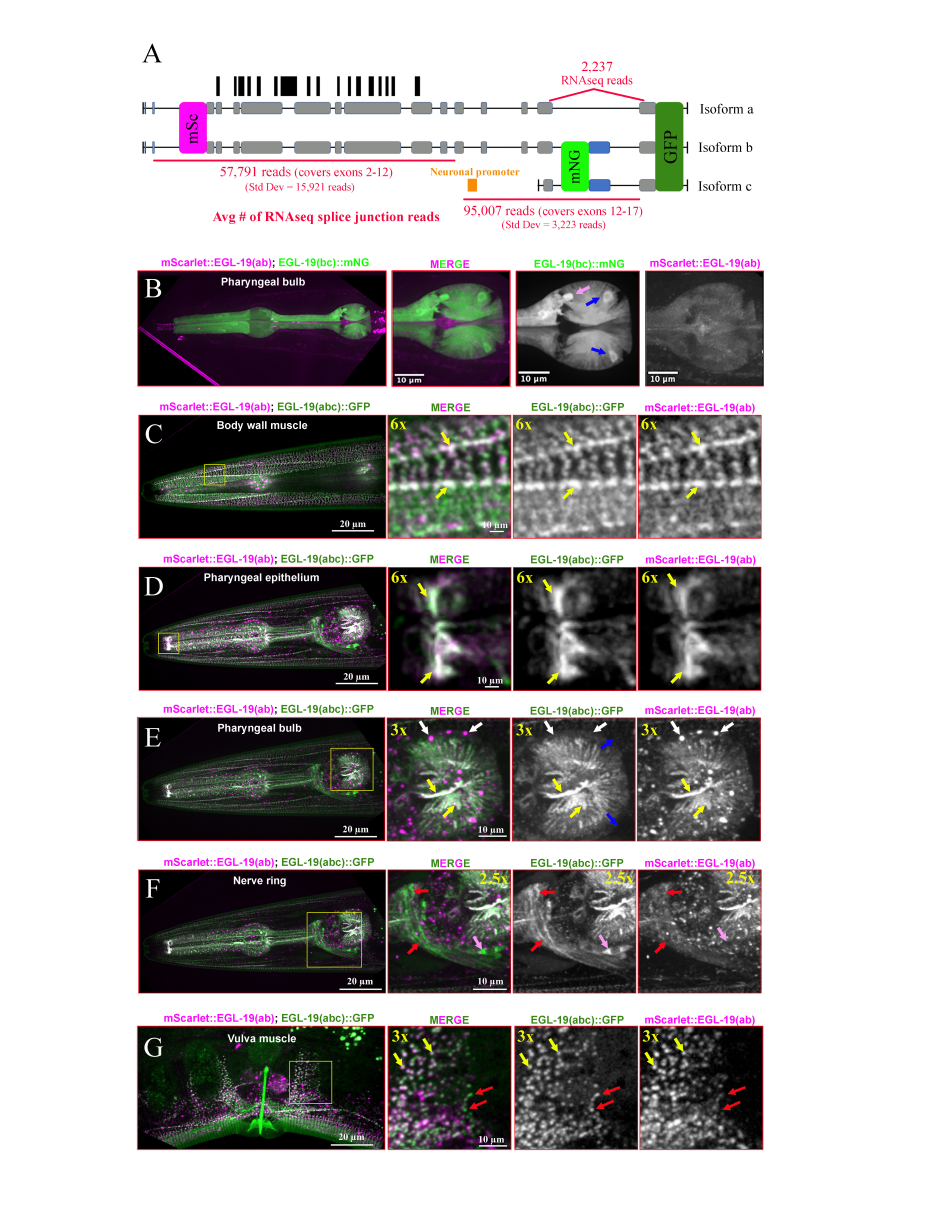
*(A)* The *egl-19* locus is predicted to encode three isoforms of EGL-19 (a, b, and c). Exons are indicated as gray boxes linked by introns. The exon highlighted blue is unique to isoforms b and c. Black boxes represent the locations of the regions encoding the transmembrane domains that form the ion channel pore. Note that the truncated c isoform lacks these transmembrane domains. Fluorescent tags were inserted using CRISPR/Cas9 knock-in at the endogenous *egl-19* locus. Colored boxes indicate the insertion location for each fluorescent tag relative to *egl-19* gene structure (mSc = mScarlet and mNG = mNeonGreen). Orange box indicates the putative location of a neuron-specific promoter identified by the RegAtlas project (Serizay *et al* . 2020). Red font refers to RNAseq splice junction data that was obtained from WormBase (version WS288) and analyzed in this study. *(B-G)* Confocal imaging of EGL-19 tag expression. Yellow arrows indicate overlapping expression of mScarlet and GFP, revealing localization of the EGL-19 (b) isoform. Red arrows indicate expression of GFP, revealing localization of the EGL-19 (c) isoform. White arrows indicate punctate expression of mScarlet, which we hypothesize represents accumulation of mScarlet:: EGL-19 in lysosomes. Pink arrows refer to a neuron which expresses both mNG and GFP, suggesting robust expression of EGL-19 (c). Blue arrows refer to a bilateral pair of neurons which express mNG, but not GFP or mScarlet, suggesting more limited expression of EGL-19 (c). *(B)* Representative images of co-expression of mScarlet:: EGL-19 (ab)/ EGL-19 (bc)::mNG in the pharyngeal bulb. Animals are F1 heterozygotes obtained from mating homozygous *mScarlet:: egl-19 (ab)* and *egl-19 (bc)::mNG* parents. mNG expression in the pharyngeal muscle is so bright that it precludes analysis of mScarlet co-localization (compare mNG in B with GFP in E). *(C-G)* Representative images of co-expression of mScarlet:: EGL-19 (ab)/ EGL-19 (abc)::GFP in several different tissues. Animals are F1 heterozygotes obtained from mating homozygous *mScarlet:: egl-19 (ab)* and *egl-19 (abc)* :: *GFP* parents. mScarlet and GFP fluorescence levels are relatively balanced, allowing observation of overlapping expression in the “MERGE” column.

## Description

EGL-19 is the sole worm ortholog of the human L-type voltage-gated calcium channel (VGCC) alpha-1C subunit, CACNA1C (Lee *et al.* 1997). CACNA1C forms an ion channel pore to allow calcium ions to enter the cytosol, which is essential for cellular calcium signaling, neuronal excitability, muscle contraction, and regulation of gene expression (Bozarth *et al.* 2018). Dysfunction of CACNA1C channels has been associated with multiple muscle and neurodevelopmental disorders, including Duchenne muscular dystrophy, Timothy syndrome, Brugada syndrome, neonatal onset severe epileptic encephalopathy, and autism spectrum disorder (Adam *et al* . 1993; Bozarth *et al.* 2018; Fukuyama *et al.* 2014; Li *et al.* 2015; Mariol and Ségalat 2001). Despite previous studies reporting several CACNA1C disease models in *C. elegans* , underlying mechanisms of these diseases remain elusive (Buddell and Quinn 2021) . Given that *CACNA1C* gene structure is very complex (18 potential isoforms according to UniProt), the relative simplicity of *egl-19* gene structure (3 potential isoforms) makes it amenable to analysis of isoform-specific expression. *C. elegans* also allows detailed characterization of *egl-19* expression in all tissues due to its transparent body. Thus, here we have combined genetic and cellular approaches to characterize the expression pattern and relevance of each EGL-19 isoform.

The *egl-19* locus encodes three putative isoforms (a, b, and c) ( Fig. 1A ). EGL-19 (b) contains 1,877 amino acids and is most similar to CACNA1C proteins in other organisms. The similarly long EGL-19 (a) isoform omits one exon present near the C-terminus of isoform b, resulting in a protein of 1,783 amino acids ( Fig. 1A ). However, there are reasons to doubt the existence and/or relevance of this alternatively-spliced isoform. First, based on RNAseq analysis of *egl-19* splice junctions, the *egl-19 (a)* variant is expressed at just 2% of the level of *egl-19 (b)* and *egl-19 (c)* ( Fig. 1A ). Second, BLAST searches indicate that the EGL-19 (a) isoform is not found in any other nematode except *C. briggsae* , and the *C. briggsae* sequence is a predicted CDS based on the *C. elegans* gene model (E. Jorgensen, personal communication). Thus, EGL-19 (a) could represent a cDNA artifact and/or the protein could prove difficult to detect in localization studies.

Curiously, both *CACNA1C* and *egl-19* appear to encode truncated isoforms that are initiated from an alternative promoter, resulting in expression of a protein lacking the transmembrane domains that form the ion channel pore ( Fig. 1A ). Although humans have five putative truncated isoforms of CACNA1C – ranging from 125 to 462 amino acids – EGL-19 (c) is the only truncated isoform in *C. elegans* , consisting of 240 amino acids corresponding to the C terminus of EGL-19 (b) ( Fig. 1A ). There is a known motif critical to calcium signaling at the C-terminus of CACNA1C, so the truncated isoform could potentially participate in autoregulation, gene regulation, or other tissue-specific processes related to calcium signaling (Dolmetsch, 2001) . EGL-19 (c) is not conserved in any other nematodes, but there are reasons to believe that it is a genuine, expressed protein. First, there is a predicted neuron-specific promoter upstream of *egl-19 (c)* within intron 12 of *egl-19 (b)* ( Fig. 1A ) (Serizay *et al* . 2020). Second, analysis of the average number of RNAseq reads upstream versus downstream of this promoter revealed that there are nearly twice as many reads from the C-terminal end ( Fig. 1A ). This suggests that the C terminus of EGL-19 is more highly expressed than the N terminus, perhaps due to transcription of *egl-19 (c)* .

To distinguish the expression pattern of each isoform, we knocked-in red or green fluorescent protein reporter genes at different locations within the endogenous *egl-19* gene using CRISPR/Cas9 gene editing. Three distinct reporters were used to target the isoforms differentially ( Fig. 1A ). First, within the shared N terminal region of isoforms a and b, the third exon was chosen for an internal insertion of mScarlet. The third exon had the most convenient gRNA target, and this insertion was both upstream of the region encoding the first transmembrane domain and downstream of the signal peptide. Thus, this insertion site was unlikely to disrupt EGL-19 function. Here, we refer to the corresponding reporter proteins as mScarlet:: EGL-19 (ab). Second, the penultimate and unique exon shared by isoforms b and c was tagged with mNeonGreen (mNG) and the relevant proteins are referred to as EGL-19 (bc)::mNG. Finally, a third reporter was created that tags the C terminus of all three isoforms with GFP, producing a set of proteins we call EGL-19 (abc)::GFP. Overall, we hypothesized that this combination of tagging strategies would allow us to determine the expression pattern of each isoform via subtractive reasoning. In particular, we were interested in imaging *mScarlet:: egl-19 (ab) / egl-19 (bc)::mNG* heterozygotes. We reasoned that mScarlet expression would represent EGL-19 (a), mNG expression would represent EGL-19 (c), and overlapping expression would represent EGL-19 (b). Unfortunately, mNG expression was so bright that it precluded unambiguous co-localization of mScarlet ( Fig. 1B ). mNG was observed primarily in pharyngeal muscle and a few neurons, but not body wall or vulva muscles. Given that mNG expression represents both EGL-19 (b) and EGL-19 (c) [i.e. *egl-19 (bc)::mNG* ] and EGL-19 (b) is ubiquitously expressed in all muscles based on functional analysis of *egl-19* , this suggests that internal insertion of mNG at the penultimate exon results in expression artifacts. One possibility is that the synthetic introns within mNG alter splicing and/or stability of the mRNAs. Another possibility is that the change in gene structure disrupts the spacing of enhancers. Indeed, there is a robust enhancer of muscle expression upstream of the mNG insertion, which could account for the very bright pharyngeal muscle expression (Serizay *et al* . 2020; B. Mueller, personal communication). Overall, this strategy unfortunately proved inconclusive for examining isoform-specific expression.

Thus, we next investigated expression in *mScarlet:: egl-19 (ab)* / *egl-19 (abc)::GFP* heterozygotes. In this scenario, overlapping expression would represent both EGL-19 (a) and EGL-19 (b), whereas GFP expression alone would represent EGL-19 (c). This would at least allow us to confirm the existence of the EGL-19 (c) protein. We were able to make several observations from this analysis. First, not surprisingly, *egl-19* is expressed in all muscle types ( Fig. 1C, E, and G ). Second, every type of muscle, several neurons, and the neuropil of the nerve ring show expression of GFP with no overlapping mScarlet, indicating that EGL-19 (c) is expressed ( Fig. 1C -G). This is consistent with the increased number of RNAseq reads at splice junctions representing the C terminus ( Fig. 1A ). Third, within muscle cells, the EGL-19 isoforms appear to be expressed in distinct subcellular localizations ( Fig. 1C, E, and G ). However, we believe that some of this expression is artifact, particularly instances where mScarlet expression alone is observed. This implies that mScarlet and GFP can be uncoupled, despite the fact that both EGL-19 (a) and EGL-19 (b) are tagged with both reporters. mScarlet is known to be acid tolerant, making it resistant to lysosomal degradation (Shinoda *et al.* , 2018). Thus, a plausible explanation for the punctate mScarlet observed in the pharyngeal bulb is that mScarlet-tagged proteins are redirected to the lysosome where they accumulate and continue to fluoresce ( Fig. 1E ) (E. Jorgensen, personal communication). Finally, the long and short isoforms of EGL-19 appear to have distinct subcellular localizations. Indeed, the pore-forming ion channel protein EGL-19 (b) is predicted to localize to the plasma membrane (sarcolemma), whereas the soluble EGL-19 (c) isoform should localize to the cytoplasm or perhaps a specific subcellular domain. This seems particularly true in body wall muscle ( Fig. 1C ). A summary of observed *egl-19* construct expression can be found in the extended data file.

Overall, we have learned several important things from this study. First, seemingly straightforward and logical strategies for analyzing isoform-specific expression of a gene of interest can be undermined by complex gene architecture. Second, it appears that one must be cautious when interpreting expression of endogenous tags targeting internal exons. Our data suggests that this can compromise spacing of *cis* -regulatory elements and/or mRNA splicing, resulting in expression artifacts. Finally, it is likely that EGL-19 (b) and EGL-19 (c) are legitimate and functionally distinct isoforms, whereas we did not find any evidence that the EGL-19 (a) isoform is expressed.

## Methods

***Creation of CRISPR knock-in strains***

CRISPR/Cas9 knock-in was performed as previously described in detail (DeMott *et al.* 2021; Dickinson *et al* . 2015; Huang *et al.* 2021; Witten *et al* . 2023).

(1) AG463 *egl-19 (av264[ egl-19 (abc)::GFP]) IV*

Referred to herein as EGL-19 (abc)::GFP. C-terminal GFP tag of all isoforms of EGL-19 . crRNA: 5’ AAGAATCTTCTGGAAGATGA 3’; Repair template fwd primer: 5’ GATCACAAGAAGATCTACTTTTAGTTACAACTCTTGGAGCATCGGGAGCCtcagg 3’; Repair template rev primer: 5’ gttttttttgtggtgagaagaatcttctggaagatgatagttcTCACTTGTAGAGCTCGTCCATTC 3’. The repair template was amplified from plasmid pDD282. Strain available by request.

(2) GLW53 *egl-19 (utx45[ egl-19 (ab1-33)::mScarlet::3xMyc:: egl-19 (ab34-)] IV*

Referred to herein as mScarlet:: EGL-19 (ab). Internal mScarlet tag at the 5’ end of exon 3 that tags isoforms a and b. The insertion was verified by PCR and fluorescence. Left flank: 5’ ttatttgaatgagcaaaaaataaatttcag 3’; Right flank: 5' GCCGCAGTGGCAGCTTCATCATCACAAGAT 3’ (1 silent mutation); gRNA: TTGTGATGATGAAGCTGCCA; Cas9/sgRNA plasmid: pGLOW69; mScarlet^SEC^3xMyc plasmid: pGLOW60. Strain available from the CGC.

(3) GLW61 *egl-19 (utx49[ egl-19 (bc)::mNeonGreen::3xFlag:: egl-19 (bc)] IV*

Referred to herein as EGL-19 (bc)::mNG. Internal mNeonGreen tag at the 5’ end of exon 2 (isoform c) or equivalent exon 16 (isoform b) that tags isoforms b and c. The insertion was verified by PCR and fluorescence. Left flank: 5’ gaatctgcgacagcacgttg 3’; Right flank: 5’ CGTCGGCGTACctgaagatt 3’; gRNA: GAAGAGCAATGGATGAGAAG; Cas9/sgRNA plasmid: pGLOW40; mNG^SEC^3xFlag plasmid: pGLOW107. Strain available by request.

***Confocal imaging***

We captured images using a spinning disk confocal system equipped with a Nikon 60X 1.2 NA water objective, a Yokogawa CSU-X1 confocal scanning unit, and a Photometric Prime 95B EMCCD camera. All images were acquired and analyzed using Nikon’s NIS imaging software. The final version of image data was processed using the ImageJ/FIJI Bio-formats plugin (National Institutes of Health) (Linkert *et al* . 2010; Schindelin *et al* . 2012).

## Extended Data

Description: Summary of egl-19 construct expression. Resource Type: Dataset. DOI: 10.22002/mfrv8-kww12
